# Primary ovarian cancer chemotherapy: current standards of care

**DOI:** 10.1038/sj.bjc.6601494

**Published:** 2003-12-17

**Authors:** W P McGuire, M Markman

**Affiliations:** 1 Franklin Square Hospital Center, Baltimore, MD 21237, USA; 2 Cleveland Clinic Foundation, Cleveland, OH 44195, USA

**Keywords:** ovarian cancer, chemotherapy, treatment

## Abstract

Chemotherapy has been regarded as standard therapy for the majority of women with advanced epithelial ovarian cancer for several decades, with this role filled largely by the alkylating agents — used as monotherapy — until the mid-1980s. The activity of cisplatin in this disorder was established during the 1970s, and combinations of cisplatin and an alkylating agent were widely used during the late 1980s. However, further research prompted by continuing concerns over poor survival and tolerability led to the adoption of paclitaxel in combination with either cisplatin or carboplatin as first-line therapy in ovarian cancer during the 1990s. Most recent research has focused on further optimisation of these regimens to maximise clinical benefit while minimising toxicity, and investigations into alternative taxanes (e.g. docetaxel), other novel agents and new treatment schedules are ongoing.

## EPIDEMIOLOGY AND DISEASE SYMPTOMS

Despite advances in treatment over the last 40 years, ovarian cancer is the second most commonly diagnosed gynaecological malignancy, and causes more deaths than any other cancer of the reproductive system. Over 25 400 new cases and 14 300 deaths were expected in the USA alone in 2001 (American Cancer Society, 2003).

Epithelial ovarian cancer is the most common histological type: at least 80% of tumours arise from the coelomic epithelium, of which 75% are serous crystadenocarcinomas. Other less common types include mucinous, endometroid, transitional cell, Brenner, clear cell and unclassified carcinomas. The remaining 20% are germ-cell and sex cord-stromal cell tumours, and those associated with metastatic spread to the ovaries (Beers and Berkow, 1999).

Ovarian cancer is not easily diagnosed because the most common presenting symptoms of persistent abdominal distension — pain and pressure in the pelvis — can be attributed to a number of causes (Lister-Sharp *et al*, 2000). Patients may be asymptomatic until an abdominal mass is discovered during routine pelvic examination or until the tumour has metastasised (Memarzadeh and Berek, 2001); consequently, progression to late stage before diagnosis is seen in the majority of presenting women. Approximately 75% of patients are at International Federation of Gynecology and Obstetrics (FIGO) stages II–IV at the time of diagnosis (Beers and Berkow, 1999; Lister-Sharp *et al*, 2000).

In women with low-risk stage I epithelial ovarian cancer, 5-year survival rates can be as high as 90% (Memarzadeh and Berek, 2001); however, these rates fall progressively as the disease becomes more advanced (to 11% in patients with stage IV malignancy).

## SURGERY AND CHEMOTHERAPY FOR OVARIAN CANCER

Surgery is currently the intervention of first choice in ovarian cancer (Lister-Sharp *et al*, 2000). Comprehensive surgical staging is indicated if malignancy is suspected or confirmed, with omentectomy and sampling of pelvic and para-aortic lymph nodes (Beers and Berkow, 1999; National Cancer Institute (NCI), 2002). Hysterectomy with bilateral salpingo-oophorectomy is usually indicated and in young patients with low-grade unilateral epithelial lesions or nonepithelial malignancy, reproductive capability can be preserved by the excision of the affected ovary only (with completion of surgical staging procedures). However, in advanced cases, tumour debulking is recommended to improve the efficacy of adjunctive therapies (Beers and Berkow, 1999). Optimal debulking can be achieved in the majority of patients, and prognosis is directly related to the success of such cytoreductive surgery (Beers and Berkow, 1999; Memarzadeh and Berek, 2001).

**Figure 1:**
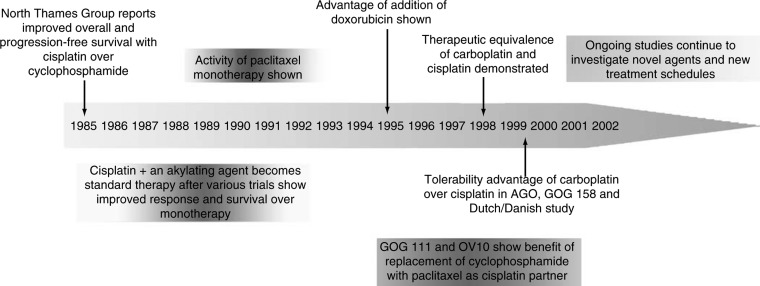
Evolution of chemotherapy for advanced ovarian cancer from the mid-1980s.

Chemotherapy for ovarian cancer has progressed considerably over the past two decades, with treatment for advanced disease moving from the use of alkylating agents to current recommended regimens based on taxanes and platinum compounds. (Dunton, 1997; Lister-Sharp *et al*, 2000; Memarzadeh and Berek, 2001). This review summarises the history of chemotherapy in ovarian epithelial cancer (major events from the mid-1980s to the present day are illustrated in Figure 1

), and discusses the development of the regimens currently used in clinical practice.

## HISTORY OF CHEMOTHERAPY

Twenty years ago, women with advanced ovarian cancer were treated most commonly with the alkylating agents melphalan, cyclophosphamide, chlorambucil and thiotepa — all as monotherapy. These drugs were associated with overall objective response rates ranging between 33 and 65%, with complete clinical responses being seen in approximately 20% of patients (Young *et al*, 1979; Dunton, 1997). The median survival among responders was approximately 17–20 months (Dunton, 1997).

A series of studies carried out from the mid-1970s onwards established cisplatin as one of the most active agents available for ovarian cancer, with Wiltshaw and Kroner (1976) reporting an overall response rate of 26.5% in 34 patients resistant to alkylating agents. Similarly, Young *et al* (1979) obtained objective responses (one of which was complete) in 29% of 25 patients refractory to alkylating agents. In 1985, the North Thames Cooperative Group reported the results of the first randomised comparison of first-line single-agent cisplatin with an alkylating agent (cyclophosphamide) in 86 women with advanced ovarian cancer, and showed significantly longer survival and response duration in patients receiving platinum therapy (Lambert and Berry, 1985). After the publication of these results and other data showing superior response rates and survival with combination over single-agent therapy (Neijt *et al*, 1984; Williams *et al*, 1985; Omura *et al*, 1986; Advanced Ovarian Cancer Trialists' Group, 2000), combinations of cisplatin with an alkylating agent became established as standard treatment.

Further analysis indicated a possible clinical benefit from the addition of an anthracycline to cisplatin–alkylating agent regimens. A meta-analysis of data from 10 trials in 1702 patients (A'Hern and Gore, 1995), five of which compared cyclophosphamide plus cisplatin (CP) with cyclophosphamide, cisplatin and doxorubicin (CAP), showed a modest — but significant — improvement in survival for the doxorubicin regimens (overall hazard ratio 0.85; *P*=0.003). The potential benefit obtained from the addition of anthracyclines has since intrigued research groups, and CAP regimens are the basis of two large-scale trials discussed in later sections of this paper (International Collaborative Ovarian Neoplasm (ICON) Collaborators, 1998; ICON Group, 2002). Most investigators in the United States abandoned anthracyclines in 1986 due to concerns that the cardiotoxicity outweighed the clinical benefit.

A retrospective review reporting a significant correlation between the dose intensity of cisplatin and response rates and survival in women with ovarian cancer (Levin and Hryniuk, 1987; see McGuire, 2000 for further details) prompted a series of dose-intense chemotherapy studies. Such studies were conducted with the aim of further improving platinum-based chemotherapy and minimising the emergence of drug resistance. Overall, data from 10 trials focusing on platinum agents in nearly 2000 patients suggested improvements in outcomes with dosages of up to 25 mg m^−2^ week^−1^, with increasing toxicity but no further clinical benefit above that level observed (McGuire, 2000). Of related interest is intraperitoneal chemotherapy, which offers the potential advantage of exposing tumour cells to higher localised doses of chemotherapy than would be possible with systemic administration. The results of clinical trials carried out to date are inconclusive, however, and the place of high-dose or intraperitoneal chemotherapy in the treatment of ovarian cancer remains under investigation (McGuire, 2000; Kaye, 2001).

### The emergence of taxane-based combinations

A significant development in the search for more effective chemotherapeutic drugs in the treatment of ovarian cancer was the discovery of the taxane class. The taxanes were originally derived from the bark of the Pacific Yew tree, *Taxus brevifolia*, and paclitaxel was identified as the active constituent in 1971. Docetaxel, introduced later, is a semisynthetic taxoid derived from the needles of *T. baccata* (Lister-Sharp *et al*, 2000). These agents promote the assembly of microtubules and inhibit depolymerisation; and this action (unique to the taxanes) disturbs mitosis in normal and malignant cells (Schiff *et al*, 1979). Early studies carried out in the late 1980s and early 1990s in 70 evaluable patients showed encouraging activity (overall response rates of 25–30%) of paclitaxel against advanced refractory ovarian epithelial cancer (McGuire *et al*, 1989; Einzig *et al*, 1992).

Two randomised, controlled trials of first-line cisplatin-based dual therapy showed additional clinical benefit when cyclophosphamide was replaced by paclitaxel. The Gynecologic Oncology Group (GOG) 111 trial studied 386 women with stage III suboptimally debulked or stage IV disease (McGuire *et al*, 1996), whereas the Intergroup OV10 trial had wider selection criteria and assessed 675 women with FIGO stage IIb, IIc, III or IV disease with or without successful debulking (Piccart *et al*, 2000). Patients in both studies had received no prior radio- or chemotherapy. Patients in GOG 111 received cisplatin 75 mg m^−2^ plus paclitaxel 135 mg m^−2^ over 24 h or cyclophosphamide 750 mg m^−2^ every 3 weeks for a total of six courses. The same drugs were compared in OV10, except that paclitaxel 175 mg m^−2^ was infused over 3 h and up to nine 3-weekly cycles were given. The median follow-up periods were 37 and 38.5 months in the GOG 111 and OV10 studies, respectively.

As shown in Table 1

**Table 1:** Clinical response and survival in the GOG 111^a^ and OV10^b^ studies

	**No. of patients evaluable for clinical response**		**Overall response rate (%)**		**Complete response rate (%)**		**Median progression-free survival (months)**		**Overall median survival (months)**	
**Treatment arm**	**GOG 111**	**OV10**	**GOG 111**	**OV10**	**GOG 111**	**OV10**	**GOG 111**	**OV10**	**GOG 111**	**OV10**
Cisplatin+paclitaxel	100	162	73	58.6*	51*	40.7*	18*	15.5*	38*	35.6*
Cisplatin+cyclophosphamide	116	161	50	44.7	31	27.3	13	11.5	24	25.8

a Cisplatin 75 mg m^−2^+either paclitaxel 135 mg m^−2^ over 24 h or cyclophosphamide 750 mg m^−2^ every 3 weeks (McGuire *et al*, 1996).

b Cisplatin 75 mg m^−2^+either paclitaxel 175 mg m^−2^ over 3 h or cyclophosphamide 750 mg m^−2^ every 3 weeks (Piccart *et al*, 2000).

* Statistically significant difference between treatments (*P*<0.05).

, both studies showed statistically significant improvements in the median overall and progression-free survival when paclitaxel was used in place of cyclophosphamide. Overall survival improvements were particularly impressive, with paclitaxel-treated patients surviving for a median 10–14 months longer than those who received cyclophosphamide. In addition, complete clinical responses were obtained with paclitaxel plus cisplatin in statistically significantly greater proportions of evaluable patients in both studies (Table 1).

However, these improvements with paclitaxel were accompanied by increased toxicity. The incidence of neutropenia, febrile neutropenia, alopecia and peripheral neurotoxicity were significantly (*P*⩽0.05) higher overall in the paclitaxel-treated group in GOG 111. While grade III/IV neutropenia and febrile neutropenia were noted, alopecia and peripheral neurotoxicity were grade II/III events. In addition, substantially more patients in the paclitaxel- than the cyclophosphamide-treated group in OV10 experienced severe myalgia, neurosensory and neuromotor symptoms, alopecia and hypersensitivity reactions. The 3-h paclitaxel infusion used in this study resulted in grade III or IV neurosensory and grade III neuromotor toxicity in 19.6 and 5% of patients, respectively, relative to 1 and 0.6% in the cyclophosphamide/cisplatin group. The levels of neurotoxicity with this 3-h infusion regimen were considerably higher than those seen with the 24-h infusion used in GOG 111 (grades III–IV neurological symptoms in 4% of patients).

### Carboplatin as a substitute for cisplatin

Cisplatin is associated with significant neurotoxicity, ototoxicity, nephrotoxicity and gastrointestinal toxicity in addition to myelosuppression, and the substantial toxicity seen in patients receiving this agent in combination with paclitaxel prompted investigations to evaluate carboplatin as an alternative taxane partner. The tolerability advantages of carboplatin rapidly became evident after its introduction in 1985; and the place of the drug in the management of ovarian cancer was solidified in 1998 by publication of a meta-analysis of 37 trials in over 5000 patients that showed (i) superiority of platinum- over nonplatinum-based treatment and (ii) equivalent efficacy of cisplatin and carboplatin (Aabo *et al*, 1998).

The addition of carboplatin rather than cisplatin to a taxane was expected to result in reductions in the incidence and severity of emesis and neurotoxicity — possibly with increased levels of myelosuppression. Accordingly, regimens containing carboplatin and paclitaxel were generally better tolerated than cisplatin plus paclitaxel in three major studies in which the two doublets showed similar efficacy (Table 2

**Table 2:** Clinical response and survival in studies comparing 3-weekly paclitaxel plus cisplatin with paclitaxel plus carboplatin. Final results of the Dutch/Danish study,^a^ the AGO^b^ and GOG 158^c^ trials

	**No. of patients evaluable for clinical response**		**Overall response rate (%)**		**Complete response rate (%)**		**Median progression-free survival (months)**			**Overall median survival (months)**		
**Treatment arm**	**Dutch/Danish**	**AGO**	**Dutch/Danish**	**AGO**	**Dutch/Danish**	**AGO**	**Dutch/Danish**	**AGO**	**GOG 158**	**Dutch/Danish**	**AGO**	**GOG 158**
Paclitaxel+cisplatin	65	75	62	81.4	35	38.7	16	19.1	19.4	30	44.1	48.7
Paclitaxel+carboplatin	67	99	66	67.7	40	31.3	16	17.2	20.7	32	43.3	57.4

a Paclitaxel 175 mg m^−2^ over 3 h+either cisplatin 75 mg m^−2^ or carboplatin to AUC 5 (Neijt *et al*, 2000).

b Paclitaxel 185 mg m^−2^ over 3 h+either cisplatin 75 mg m^−2^ or carboplatin to AUC 6 (du Bois *et al*, 2003).

c Paclitaxel 135 mg m^−2^ over 24 h+cisplatin 75 mg m^−2^ or paclitaxel 175 mg m^−2^ over 3 h+carboplatin to AUC 7.5 (Ozols *et al*, 2003).

). The Dutch/Danish study (Neijt *et al*, 2000) in 208 patients and the Arbeitsgemeinschaft Gynaekologische Onkologie (AGO) study (du Bois *et al*, 2003) in 798 patients compared 3-weekly paclitaxel 175 or 185 mg m^−2^ infused over 3 h plus cisplatin 75 mg m^−2^ with the same dosage of paclitaxel plus carboplatin infused to achieve AUC 5 or 6. Women in both studies had stage IIb–IV disease and were followed up for a median of 37 months (Neijt *et al*, 2000) or a mean of 49–50 months (du Bois *et al*, 2003). The GOG 158 trial in 792 eligible patients with optimal stage III disease compared paclitaxel 135 mg m^−2^ infused over 24 h plus cisplatin 75 mg m^−2^ with paclitaxel 175 mg m^−2^ over 3 h plus carboplatin to AUC 7.5 (Ozols *et al*, 2003).

**Figure 2:**
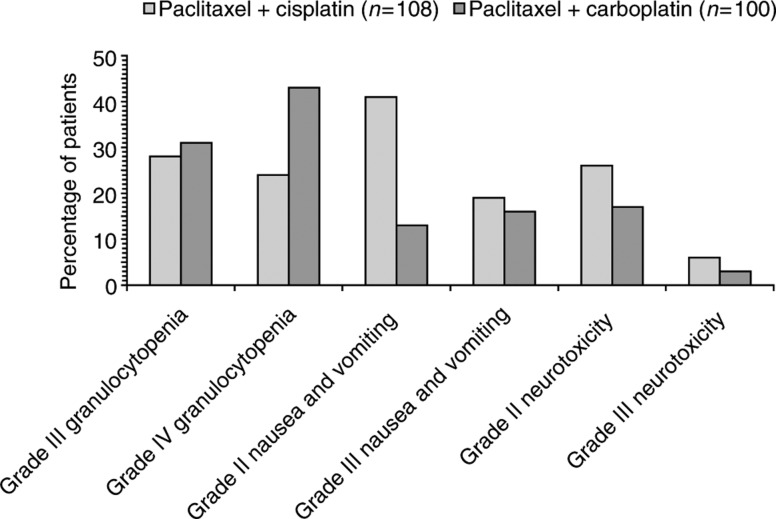
Incidence of adverse events showing differences between treatment arms in the Dutch/Danish study of 3-weekly paclitaxel 175 mg m^−2^ infused over 3 h plus either cisplatin 75 mg m^−2^ or carboplatin infused to achieve AUC 5 (Neijt *et al*, 2000).

The final results from AGO, GOG 158 and the Dutch/Danish study showed little difference between treatments in the median progression-free survival (see Table 2 for summary of available data). Although the median overall survival was similar between treatment arms in each study, it was higher among patients in the AGO and GOG 158 studies — ranging between 44 and 57 months — compared with 30 months of the Dutch/Danish study. Toxicity profiles were mainly as expected, with paclitaxel plus carboplatin being better tolerated overall. The Dutch/Danish investigators (Neijt *et al*, 2000) reported more grade III or IV granulocytopenia with paclitaxel plus carboplatin than with paclitaxel plus cisplatin, but nonhaematological toxicities — in particular neurotoxicity — were less frequent with carboplatin (Figure 2

). Patient numbers in this study were too small for definitive conclusions to be drawn, but the larger AGO study (du Bois *et al*, 2003) also showed more frequent but statistically nonsignificant haematological toxicity with carboplatin and more nonhaematological toxicity with cisplatin (grades III–IV peripheral neuropathy in 8% of patients in the carboplatin arm and in 19% of cisplatin recipients). To date, more grade IV leucopenia, grades III–IV gastrointestinal toxicity, fever and metabolic toxicity have been reported in GOG 158 with 24-h paclitaxel plus cisplatin than with 3-h paclitaxel plus carboplatin, with more thrombocytopenia and pain (probably due to paclitaxel-associated arthralgias) in carboplatin recipients as well as a statistically greater incidence of grade III/IV thrombocytopenia with the carboplatin doublet, where the AUC of carboplatin was escalated to 7.5 (Ozols *et al*, 2003).

### The optimal taxane−platinum regimen

The encouraging results obtained to date with taxane–platinum regimens have prompted further research to resolve outstanding issues; several trials, including GOG 132 (Muggia *et al*, 2000) and the second and third International Collaborative Ovarian Neoplasm group studies (ICON-2; ICON-3), have provided further insight (ICON Collaborators, 1998; ICON Group, 2002).

GOG 132 was a three-arm trial of 3-weekly paclitaxel 135 mg m^−2^ over 24 h plus cisplatin 75 mg m^−2^ compared with high-dose cisplatin (100 mg m^−2^) or paclitaxel (200 mg m^−2^ over 24 h) alone, each for six cycles, in 614 patients (Muggia *et al*, 2000). As shown in Table 3

**Table 3:** Summary of clinical results from the GOG 132 study of 3-weekly paclitaxel 135 mg m^−2^ over 24 h plus cisplatin 75 mg m^−2^ compared with cisplatin alone (100 mg m^−2^) or paclitaxel alone (200 mg m^−2^ over 24 h), each for six cycles (Muggia *et al*, 2000)

**Treatment arm**	**No. of patients evaluable for clinical response**	**Overall response rate (%)**	**Complete response rate (%)**	**Median progression-free survival (months)**	**Median overall survival (months)**
Cisplatin	122	67*	42	16.4*	30.2
Paclitaxel	131	42*	21	10.8*	25.9
Cisplatin+paclitaxel	124	67*	43	14.1*	26.3

* Statistically significant difference between treatments (*P*<0.05).

, the results showed no significant difference in the median overall survival among treatment arms; however, cisplatin alone or in combination yielded superior response rates and progression-free survival. In addition, the combination of paclitaxel and cisplatin was better tolerated overall than cisplatin alone. Neutropenia, fever and alopecia were more frequent and severe with the paclitaxel regimens than with cisplatin alone (*P*<0.001), and neutropenia (*P*=0.008) and febrile episodes (*P*<0.001) tended to be more severe with paclitaxel monotherapy than with the combination regimen. Not only were anaemia, thrombocytopenia and gastrointestinal toxicity more frequent and severe (*P*<0.001) in the cisplatin groups, but renal toxicity was also most severe (*P*<0.001) and there was a tendency for more frequent and severe neurotoxicity in the cisplatin monotherapy group. Both monotherapies were discontinued more frequently than the combination: 17% of patients withdrew from cisplatin treatment resulting from toxicity or patient refusal *vs* 7% in the combination group, and 20% withdrew from paclitaxel monotherapy because of early disease progression compared with 6% in the combination group.

It was suggested that the similarity in overall survival across treatment arms may have been related to the frequency of treatment crossover in this study, particularly from cisplatin monotherapy to paclitaxel. The similarity between results in the initial cisplatin and combination arms suggests that sequential therapy may confer benefit. No conclusions in this respect could be drawn; however, since GOG 132 was not designed or powered to show such an effect. Further studies will be needed to clarify this point. The authors concluded that on the basis of these results, taxane/platinum combination therapy should remain the preferred first-line option in advanced ovarian cancer.

**Figure 3:**
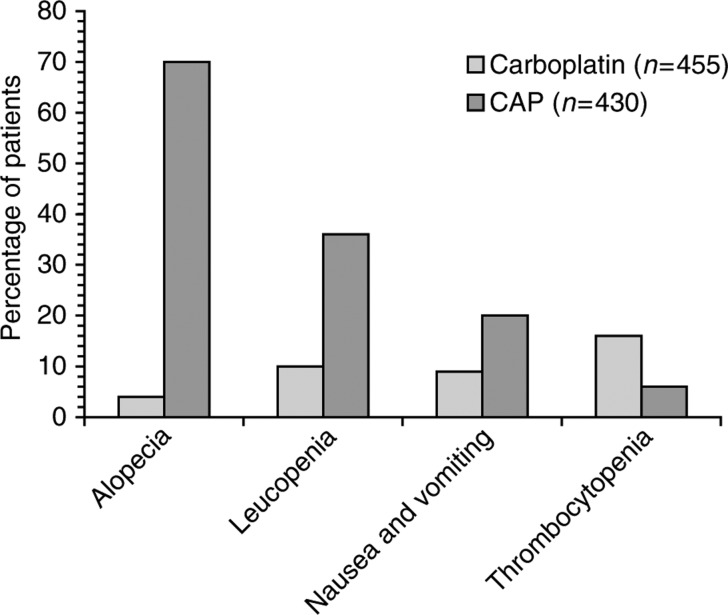
Grade III and IV toxicities reported with >5% incidence in 885 Italian patients participating in the ICON-2 comparison of 3-weekly carboplatin monotherapy (to achieve AUC 5) with cyclophosphamide 500 mg m^−2^, doxorubicin 50 mg m^−2^ and cisplatin 50 mg m^−2^ (CAP), both for six cycles, in 1526 patients from 132 hospitals (ICON Collaborators, 1998).

If sequential therapy involving taxanes and platinums is to be developed, the ICON trials provide interesting data, suggesting that single-agent carboplatin should be considered in this setting. ICON-2 compared 3-weekly carboplatin monotherapy (to achieve AUC 5) with a CAP regimen comprising cyclophosphamide 500 mg m^−2^, doxorubicin 50 mg m^−2^ and cisplatin 50 mg m^−2^, both for six cycles, in 1526 patients from 132 hospitals (ICON Collaborators, 1998). There was no difference in survival between the two groups (median progression-free survivals was 15.5 and 17 months for carboplatin and CAP, respectively, with a median overall survival of 33 months in both groups), and there was no evidence of any difference in efficacy in any subgroup of patients (e.g. age, FIGO stage, residual tumour bulk and histology). However, CAP was substantially more toxic than carboplatin, causing more alopecia, leucopenia and nausea (detailed toxicity data were available for patients attending Italian centres only; major events are shown in Figure 3

).

ICON-3 can be viewed as two parallel randomised trials comparing carboplatin alone with carboplatin plus paclitaxel, and carboplatin plus paclitaxel with CAP in a total of 2074 patients (ICON Group, 2002). Paclitaxel was given at a dose of 175 mg m^−2^ by 3-h infusion but depending on the method used to determine glomerular filtration rate (GFR), the carboplatin dose was a minimum of either AUC 5(GFR+25) or 6(GFR+25) mg. All regimens were administered every 3 weeks for up to six cycles. The final results, for a median follow-up of 51 months, showed no significant differences in overall survival between carboplatin plus paclitaxel and carboplatin alone or CAP (hazard ratio 0.98; *P*=0.74), which does not appear to concur with the positive results reported for cisplatin plus paclitaxel in GOG 111 (McGuire *et al*, 1996) and OV10 (Piccart *et al*, 2000). However, ICON-3 recruited a wide range of patient types — disease stages I–IV, with 46% of patients with residual tumour bulk ⩾2 cm, 30% with no or microscopic disease and 55% with poorly differentiated disease (ICON Group, 2002). Although no statistically significant differences were seen in patients treated with or without paclitaxel, an early trend towards overall survival benefit was noted in favour of paclitaxel plus carboplatin, from between 12 and 36 months from randomisation in patients with residual tumour bulk ⩾2 cm. It was noted that approximately one-third of patients in the control group went on to receive a taxane at some stage (with or without platinum), mainly after disease progression. Therefore, it seems possible that the efficacy of taxanes and platinum agents, together or as monotherapy, may depend at least in part on the manner in which they are sequenced, and it may be desirable to investigate and specify further the optimum way in which to use the taxanes.

### Taxanes as continuation therapy

Of additional interest is the observation of the prolongation of progression-free survival with the use of continuation therapy with single-agent paclitaxel after complete response to platinum/paclitaxel therapy (Markman *et al*, 2002). A total of 277 patients with advanced ovarian cancer were randomised to either 3 or 12 months follow-on treatment with paclitaxel (initially 175 mg m^−2^ every 28 days, subsequently reduced to 135 mg m^−2^ because of concerns regarding a higher drop-out rate in the 12-month arm).

The improvement in the 12-month arm was sufficiently compelling for this trial to be terminated early. The median progression-free survival in the 3- and 12-month groups was 21 and 28 months, respectively (*P*=0.0023 by adjusted Cox model analysis), with a 3- *vs* 12-cycle progression hazard ratio of 2.31. However, there was no significant difference between groups in the median overall survival at the date of study closure.

## CONCLUSIONS

For several decades, chemotherapy has been the mainstay of treatment in all but early-stage and well-differentiated malignant ovarian tumours; the literature shows a clear progression from a dependence on alkylating agents to the platinum-based regimens in use today. Results of numerous randomised-controlled trials have pointed to taxane–platinum combinations as the standard of care for women with advanced ovarian cancer, and these are now generally recommended for first-line treatment (Lister-Sharp *et al*, 2000). There is currently no call for a change in this recommendation (Tattersall, 2002), although the availability of mature data from the ICON-3 trial offers an opportunity for review and refinement of treatment guidelines.

Despite the progress discussed in this review, most patients with advanced ovarian cancer eventually die from their disease. Further improvements in toxicity, response rates and survival may result from the use of an alternative taxane such as docetaxel, from the incorporation of other agents or from the use of different treatment schedules. Agents of particular interest in this respect include topotecan, gemcitabine, epirubicin, liposomal doxorubicin, etoposide and oxaliplatin, and it is expected that ongoing research will contribute to the improvement of outcomes as the chemotherapy of advanced ovarian cancer continues to develop.
